# Alcohol as a risk factor for hearing loss: A systematic review and meta-analysis

**DOI:** 10.1371/journal.pone.0280641

**Published:** 2023-01-20

**Authors:** Peiyi Qian, Zhixin Zhao, Shuangyan Liu, Jiarui Xin, Yun Liu, Yinzhu Hao, Yaxin Wang, Lei Yang

**Affiliations:** 1 School of Public Health, Hangzhou Normal University, Hangzhou, Zhejiang, China; 2 Hangzhou Hospital for the Prevention and Treatment of Occupational Disease, Hangzhou, Zhejiang, China; 3 School of Public Health, Tongji Medical College, Huazhong University of Science and Technology, Wuhan, Hubei, China; 4 National Institute of Occupational Health and Poison Control, Chinese Center for Disease Control and Prevention, Beijing, China; Shahrood University of Technology, ISLAMIC REPUBLIC OF IRAN

## Abstract

**Objective:**

Growing evidence suggests that alcohol consumption is a risk factor for hearing loss; however, the evidence has been inconsistent. This systematic review and meta-analysis aimed to evaluate the effect of alcohol consumption on hearing loss.

**Methods:**

We searched several databases up to November 2021, for published articles using the keywords “alcohol drinking” and “hearing loss”. Two investigators independently conducted the study selection and data extraction. Based on the results of the heterogeneity analysis (*Q* statistic and *I*^2^ statistic), a fixed- or random-effects model was used to calculate the pooled odds ratios (ORs). Subgroup and sensitivity analyses were performed to assess the potential sources of heterogeneity and robustness of the pooled estimation. Publication bias in the literature was evaluated using Egger’s test.

**Results:**

In total, 18 (9 cross-sectional, 5 case-control, and 4 cohort) observational studies were identified in this search; 27,849 participants were included. Compared with non-drinkers, the pooled OR of drinkers was 1.22 (95% confidence interval: 1.09–1.35).

**Conclusion:**

Evidence suggests a positive association between alcohol consumption and hearing loss. Drinkers were at a higher risk than non-drinkers. Drinking limitations may be useful for preventing hearing loss.

## Introduction

Hearing loss is a chronic and disabling sensory disorder and a major public health concern worldwide. The latest estimates reveal that hearing loss currently affects 1.59 billion people worldwide, which is 20.3% of the global population, of whom 430 million (5.5%) have moderate or severe hearing loss [1]. By 2050, the number of people with hearing loss is anticipated to reach nearly 2.5 billion, of whom 700 million will require interventions [2]. When unaddressed, hearing loss has an extensive negative effect on both individuals and society. According to statistics, hearing loss was ranked as the third leading cause of global years lived with disability in 2019 [2], affecting people’s quality of life, communication, cognition, education, employment, and social participation [3–7]. At the societal level, unaddressed hearing loss poses an enormous economic burden, estimated to be more than US $750 billion per year globally [8].

The occurrence and development of hearing loss is multifactorial and is a long-term and chronic process. In addition to noise exposure, other factors such as genes (*CAT*, *HSP70*, *CDH23*, *CASP*, *NOX3*, *and NRN1*) [9–12], environmental factors [13, 14], and individual lifestyles [15, 16] (smoking frequency, physical exercise, use of protective devices) may be independent factors or have a synergistic effect with noise to increase the risk of hearing loss.

Alcohol consumption is a common modifiable risk factor for many health problems, such as liver cancer and cardiovascular diseases [17–19]; additionally, its association with hearing loss has been inconclusive in previous studies. Shrestha [20] Lao [21], and Park [22] found that alcohol consumption was a risk factor for hearing loss, while Curhan’s [23] research showed that alcohol consumption was not significantly related to hearing loss. Some studies found a protective effect of proportionate drinking against hearing loss, which was explained to be similar to the cardioprotective effects of alcohol [24, 25]. These results were inconsistent, mainly owing to differences in study population, age, sex, sample size, hearing loss examination, definition of “alcohol drinking,” or other confounding factors.

Therefore, we conducted a systematic review and meta-analysis to investigate and quantify the association between alcohol consumption and hearing loss. We reduced the conceptual heterogeneity by including only observational cross-sectional, case-control, and cohort studies that assessed hearing loss using pure-tone audiometry. And, we conducted exploratory subgroup analyses to examine possible explanations for heterogeneity owing to demographics, study type, hearing loss type, and analysis factors.

## Materials and methods

### Literature search strategy

This meta-analysis was performed according to the PRISMA guidelines [26]. We carried out a literature search up to November 30, 2021, in Embase, PubMed, Web of Science, Wanfang, Weipu, and CNKI databases for studies published in English and Chinese using the Medical Subject Headings terms “alcohol drinking” and “hearing loss”, and their with related words, following the Meta-analysis of Observational Studies under Epidemiology guidelines [27]. Two reviewers independently screened the potential publication titles and reviewed the full text of the eligible articles. The review has been pre-registered with PROSPERO (CRD 42021247735).

### Inclusion and exclusion criteria

The inclusion criteria for relevant articles used in this systematic review were as follows: (1) cohort-based, case-control, or cross-sectional study; (2) studies that investigated the risk of hearing loss in drinkers; (3) studies that identified hearing loss by pure-tone average; and (4) studies that provided odds ratio (OR) or relative risk (RR) with the corresponding 95% confidence interval (CI).

The exclusion criteria were as follows: (1) studies in the format of reviews, editorials, master and doctoral dissertations, and abstracts; (2) studies with a small sample size (n <30); (3) studies whose subjects were children (<15 years old); (4) studies in which the outcome was sudden deafness; (5) studies that middle ear disease was identified by a test of tympanometry or bone conduction.

### Data extraction and quality assessment

The following information from the selected studies was independently extracted by two investigators: principal author, year of publication, study design, number of subjects, sex and age of participants, country, information about the alcohol consumption, diagnostic criteria of hearing loss, adjusted OR/RR with 95% CIs, adjusting or matching variables, and statistical analyses. Noise-Induced Hearing Loss (NIHL) [28] is caused by prolonged exposure to noise, it is characterized as sensorineural hearing loss and is usually bilateral, irreversible, and progressive while the exposure to noise continues. Age-related hearing loss (ARHL) [29] is a complex disorder that results from the cumulative effects of aging on the auditory system. It is defined as a progressive, bilateral, symmetrical age-related sensorineural hearing loss, which is most pronounced at the higher frequencies. When multiple reports were published in the same study, we only considered the most informative one in this meta-analysis. The most fully adjusted OR/RR was chosen when several estimates for the same exposure were reported with different adjustment levels.

The methodological quality of the included cross-sectional studies was assessed using the 11-item checklist recommended by the Agency for Healthcare Research and Quality. The article quality was assessed as follows: low quality = 0–3, moderate quality = 4–7, and high quality = 8–11. The quality of the included cohort and case-control studies was assessed using the Newcastle-Ottawa Scale (NOS); the rating criteria for the NOS were as follows: Low quality = 1–5, medium quality = 6–7, and high quality = 8–9 [30].

### Statistical analysis

OR was used to measure the association between alcohol consumption and hearing loss. Owing to the low incidence of hearing loss, the reported RR was approximately considered as an OR. When the OR was reported separately at different drinking levels, we extracted the highest level of results. One study separately estimated the OR and 95% CI in men and women, and it was treated as a different study in the analysis. Prior to merging the overall pooled ORs, the Q test and *I*^*2*^ test were performed to evaluate heterogeneity. When the Q test *P*-value was more than 0.10 or the *I*^*2*^ value was less than 50%, the ORs were pooled according to the fixed-effects model. Otherwise, a random effects model was applied [31]. Forest plots were generated for alcohol drinkers versus nondrinkers. Subgroup analyses were based on study design, mean age, sex, publication year, population type, hearing loss type, and region to investigate potential sources of heterogeneity. We also performed a sensitivity analysis by removing each individual study from the meta-analysis and recalculating the summary risk estimate. Moreover, we used qualitative visual inspection of funnel plots and quantitative Begg’s tests to assess publication bias for the risk of hearing loss. All reported *P* values were two-sided, and *P*<0.05 was considered statistically significant for all included studies. Statistical analyses were performed using Stata/MP version 16.0 (StataCorp, College Station, TX, USA).

## Results

### Literature research and study characteristics

**Fig 1:**
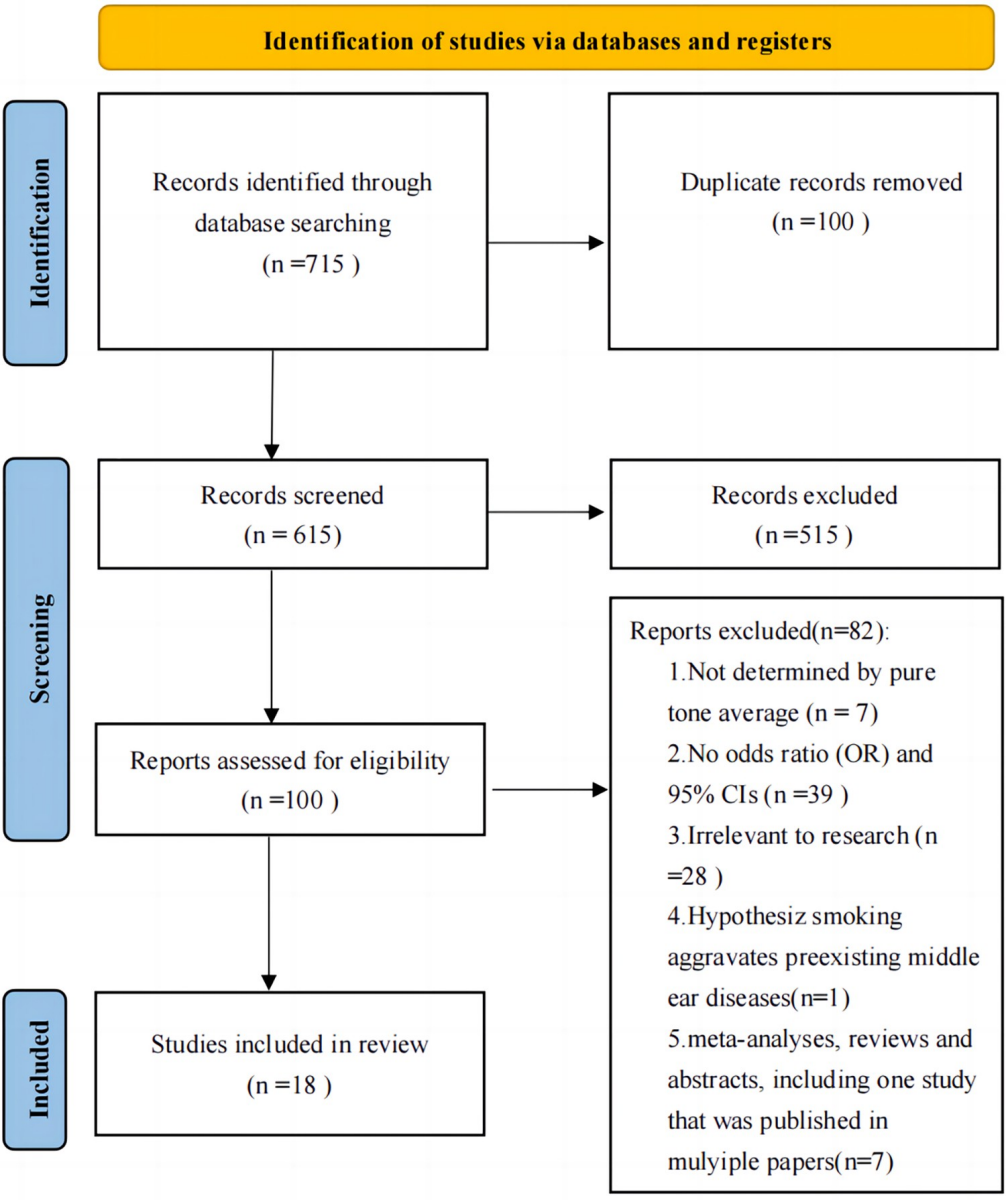
Flow chart of the selection of studies on alcohol consumption and risk of hearing loss included in the systematic review and meta-analysis. ***Note*.** Abbreviations: CI, confidence interval; OR, odds ratio.

Fig 1 presents the details of the literature search. First, 715 articles were identified. Of these, 615 articles were excluded based on the information provided in the title and abstract. After retrieving and reviewing 100 full articles, we excluded 82, of which 28 were irrelevant to our research. Seven studies were excluded because the participants had not been evaluated using pure-tone audiometry to confirm hearing loss. In addition, 39 studies did not provide an effect size estimate or 95% CIs to calculate the pooled OR. One study hypothesized that drinking aggravates preexisting middle ear diseases and was excluded. Seven meta-analyses, reviews, and abstracts were excluded. One article from Korea, from 2017 and another from 2019 used data from the same public database, the Korea National Health and Nutrition Examination Surveys (KNHANES), and the two may have had a high degree of data overlap. The methods and content of the two articles were fully compared, the article from 2019 was better and retained. Finally, 18 articles met our initial inclusion criteria.

The main characteristics of the 18 selected studies, comprising 27,849 participants, are presented in Table 1. There were four cohort studies, five case-control studies, and nine cross-sectional studies. Fourteen studies were from Asia and four were from non-Asian regions. Twelve studies included both men and women, five studies involved men, and one study involved women only. All the studies described a method for the assessment of hearing impairment. Some studies (9 studies) used 25 dB as the hearing impairment threshold, eight studies used 40 dB, and one study used 30 dB. In addition, the quality assessment scores for the four cohort studies were in the range of 6–9. For the five case-control studies, the scores were in the range of 6–8. The nine cross-sectional studies had scores ranging from 6 to 10.

**Table 1:** Main characteristics of 18 included studies on drinking and hearing loss risk.

Principal Author and Year	research type	Country	Sex	N	Age	Alcohol information	Diagnostic Criteria of HL	Statistical analysis	Adjusting or Matching Variables	Quality Assessment
Brant, 1996 [32]	Cohort	America	Men	531	>50	none, moderate, high	PTA_0.5,1,2,3_ >30dB	A proportional hazards regression model	age, smoking, alcohol consumption	7
Gopinath, 2010 [33]	Cohort	Australia	Both	2815	66.6±9.3	none, 1,>1 but ≤2, and >2 drinks/day	PTA_0.5,1,2,4_ >25dB	Logistic regression	age, sex, family history of hearing loss, occupational noise, history of diagnosed stroke, and diabetes	9
Li, 2008 [34]	Cohort	China	Men	Exposed 244/control 140	<40.0	annual alcohol consumption	PTA >25dB	Ordered multi-class logistic regression	NA	6
Guo, 2016 [35]	Cohort	China	Men	819	31.8±5.1	0, 0<n<5, 5≤n<10, 10≤n<15, ≥15	PTA_4,6,8_ >40 dB	Logistic regression	NA	7
Jiao, 2016 [36]	Case-control	China	Both	Case 187/control 187	39	drinker, non-drinker	PTA_3,4,6_ >40 dB	Conditional Logistics analysis	NA	8
Itoh, 2001 [37]	Case-control	Japan	Both	Case 496/control 2807	63.7	never, past, light, heavy	PTA_4_ >40dB	multiple logistic regression	sex, age, BMI, %VC, hemoglobin, TC, FPG, AST, ALT, and g-GTP	8
Ni, 2019 [38]	Case-control	China	Both	Case 747/Control 789	33.1	drinker, non-drinker	PTA >40 dB	Logistic regression	NA	6
Bai, 2018 [39]	Case-control	China	Both	Case 519/control 111	34.1±9.2	drinker, non-drinker	PTA >25dB	Logistic regression	NA	7
Shen, 2013 [40]	Case-control	China	Men	case 201/control 294	42.8±7.9/39.1±9.7	drinker, non-drinker	PTA_3,4,6_ >40 dB	Multiple regression	age, smoking, alcohol	7
Shrestha, 2011 [20]	Cross-sectional	Australia	Both	110	29.8	drinker, non-drinker	PTA >25dB	Chi-square test	NA	7
Popelka, 2000 [41]	Cross-sectional	America	Both	3571	65.6	never, past, current	PTA_4,6,8_ >40 dB	multiple logistic regression	age, sex, education, smoking, occupational noise exposure, alcohol consumption	9
Chen, 2020 [42]	Cross-sectional	China	Men	3655	22.3±3.1	0, 1–2, 3–4 and >5 drinks/week	PTA_3,4,6_ >25 dB	multiple logistic regression	NA	7
Zou, 2018 [43]	Cross-sectional	China	Both	808	38.5	drinker, non-drinker	PTA_3,4,6_ >25 dB	Logistic regression	NA	9
Sun, 2014 [44]	Cross-sectional	China	Men	471	39.75±6.58	drinker, non-drinker	PTA_3,4,6_ >40 dB	Logistic regression	NA	7
Gao, 2019 [45]	Cross-sectional	China	Both	389	62.7±4.9	drinker, non-drinker	PTA_0.5,1,2,4_ >40 dB	multiple logistic regression	NA	6
Park, 2019 [22]	Cross-sectional	Korea	Both	Men (1525) Women (2335)	>20.0	drinker, non-drinker	PTA_0.5,1,2,4_ >40 dB	logistic regression analyses	age	10
Xu, 2021 [46]	Cross-sectional	China	Women	1416	60.8±9.0	drinker, non-drinker	PTA_0.5,1,2,4_ >25dB	logistic regression analyses	education and SBP, older age, alcohol, consumption, and menopausal history	8
Wang, 2021 [47]	Cross-sectional	China	Both	2280	35.0±8.4	drinker, non-drinker	PTA_3,4,6_ >25dB	logistic regression analyses	sex, age, CNE, BMI, HPD usage, community noise exposure, personal earphone usage, smoking, and alcohol consumption	8

***Note*.** Abbreviations: HL, hearing loss; PTA, pure tone average; NA, non-adjusted variables; dB, decibels; SBP, systolic blood pressure; CNE, cumulative noise exposure; BMI, body mass index; HPD, hearing protection device.

### Association between alcohol consumption and risk of hearing loss

**Fig 2:**
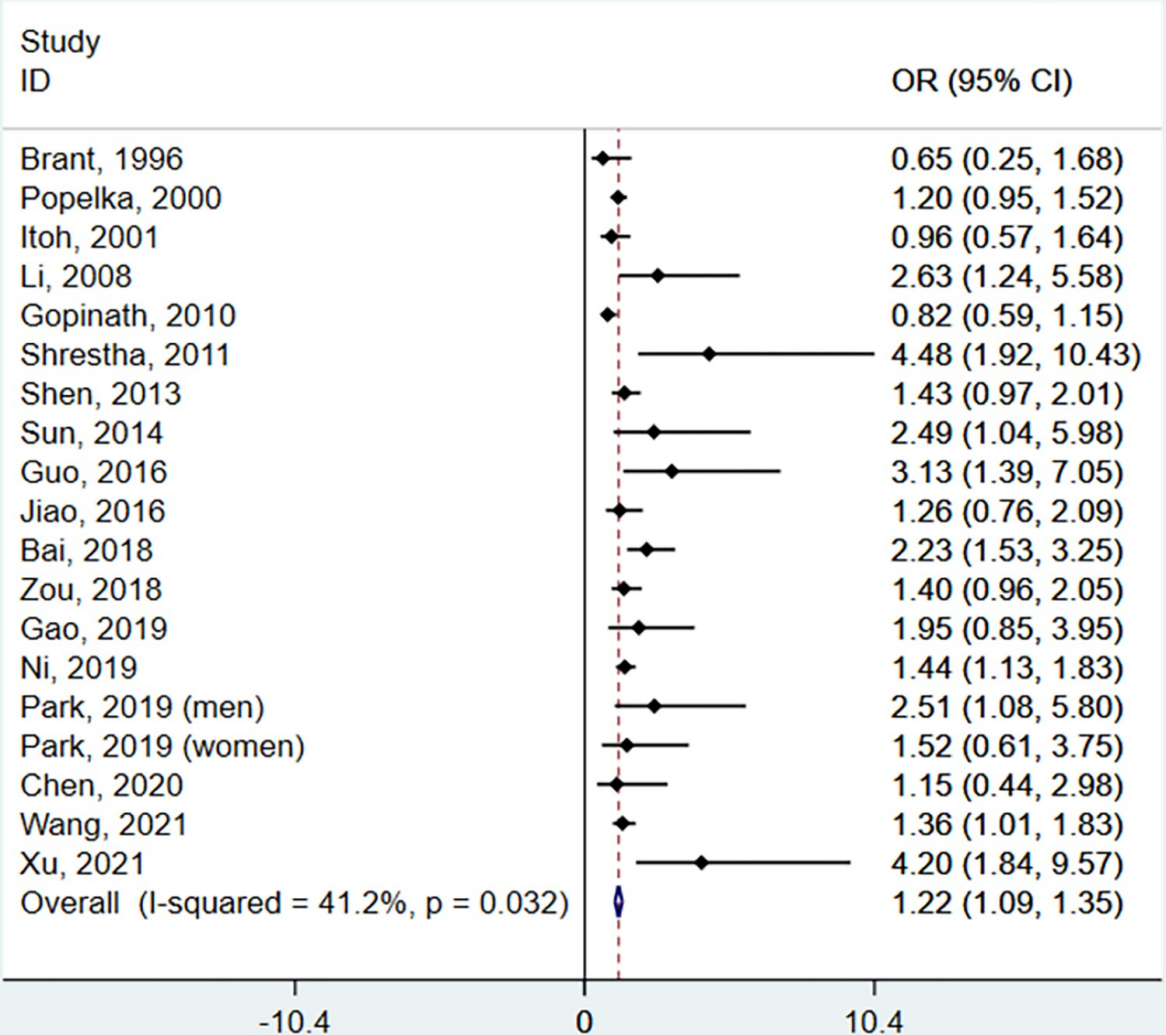
Summary estimates were calculated using fixed-effects models. ***Note*.** Abbreviations: CI, confidence interval; OR, odds ratio.

In total, 19 studies (18 plus one article that separately estimated OR and 95% CI in men and women) assessed the association between alcohol consumption and the risk of hearing loss. Among the 19 studies, nine reported a positive relationship between alcohol consumption and the risk of hearing loss, while 10 found no association. Fig 2 shows the forest plots for alcohol consumption and hearing loss risk. The pooled OR of hearing loss for drinkers was 1.22 (95% CI: 1.09–1.35), with moderate heterogeneity across studies (Q test *P* = 0.032. *I*^*2*^ = 41.2%).

### Subgroup analyses

Table 2 shows the results of the subgroup analysis for alcohol consumption and hearing loss risks. The study design, sex, mean age, publication year, population type, hearing loss type, and region were included in the subgroup analysis. Overall, the results for most subgroups indicated a positive relationship between alcohol consumption and risk of hearing loss. Our subgroup analysis revealed a statistically significant positive association between alcohol consumption and hearing loss risks, especially among studies conducted in men (OR = 1.56; 95%CI, 1.12–2.01), studies whose participants had a lower average age (OR = 1.46; 95%CI, 1.27–1.66), current studies (from 2010 onwards OR = 1.48; 95%CI, 1.28–1.67), studies of professional populations (OR = 1.41; 95%CI, 1.22–1.59), and studies conducted in Asia (OR = 1.42; 95%CI, 1.24–1.60). In addition, heterogeneity was reduced in these seven subgroups analyses.

**Table 2:** Subgroup analyses relating alcohol consumption and hearing loss.

Subgroup	Numbers of studies	Pooled OR	95% CI	*P* Value for *Q* Test	*I*^2^ (%)	*P*_heterogeneity_
**Study design**						
Cohort	4	0.84	0.59–1.10	0.142	44.9	0.004
Case-control	5	1.38	1.15–1.61	0.172	37.3	
Cross-sectional	10	1.32	1.12–1.53	0.569	0.0	
**Sex**						
Both	11	1.18	1.04–1.32	0.017	53.8	0.180
Men	6	1.56	1.12–2.01	0.559	0.0	
Women	2	1.90	0.44–3.35	0.207	37.2	
**Mean age**						
<50	11	1.46	1.27–1.66	0.530	0.0	0.001
≥50	8	1.01	0.84–1.19	0.164	33.0	
**Publication year**						
>2010	14	1.48	1.28–1.67	0.616	0.0	0.000
<2010	5	0.99	0.81–1.17	0.159	39.3	
**Population type**						
Occupation	12	1.41	1.22–1.59	0.361	8.6	0.004
Community	7	1.02	0.83–1.21	0.109	42.3	
**Hearing loss type**						
HL	7	1.30	1.03–1.57	0.347	10.8	0.000
NIHL	9	1.45	1.25–1.65	0.513	0.0	
ARHL	3	0.83	0.59–1.06	0.789	0.0	
**Region**						
Asian	15	1.42	1.24–1.60	0.501	0.0	0.005
Non-Asian	4	0.97	0.60–1.34	0.073	56.9	

***Note*.** Abbreviations: HL, hearing loss; NIHL, noise-induced hearing loss; ARHL, age-related hearing loss; OR, odds ratio; CI, confidence interval.

### Sensitivity analysis and publication bias

A sensitivity analysis was performed to investigate the source of heterogeneity. Sensitivity analysis showed that the pooled effect sizes obtained for the association of alcohol consumption with the risk of hearing loss did not depend on the individual study.

The funnel plot was used to examine publication bias in the association between alcohol consumption and hearing loss. The graph appears to be symmetrical, and a few studies fall outside the dotted line, suggesting the absence of publication bias. Moreover, we found no publication bias according to Egger’s regression test (Egger’s *P* = 0.144).

## Discussion

A meta-analysis of 18 studies published over the past 25 years showed a significant relationship between alcohol consumption and hearing loss. Compared to non-drinkers, the risk of developing hearing loss increased 1.22-fold among drinkers. Although heterogeneity was moderate in our analysis (*I*^*2*^ = 41.2%, *P* = 0.032), subgroup analysis was conducted to explore the sources of heterogeneity. The results for most subgroups indicated a positive relationship between alcohol consumption and the risk of hearing loss, and heterogeneity was reduced across these seven subgroups analyses. Sensitivity analysis showed that our results were robust, and there was no publication bias according to funnel plot and Egger’s analysis. In summary, these results suggest that alcohol consumption is an independent risk factor for hearing loss.

However, the specific mechanisms underlying alcohol consumption and hearing loss are unclear. In Curhan’s [23] prospective study, among those with lower levels of vitamin B12 intake, the consumption of higher levels of alcohol, specifically liquor (spirits), was associated with an increased risk of hearing loss. Owing to the important role of vitamin B12 in cell metabolism, vascular function, and myelin synthesis, vitamin B12 deficiency might lead to the occurrence of auditory system diseases [48]. Drinking too much alcohol depletes vitamin B12 in the liver, and this increases the possibility of hearing loss among drinkers [49]. Ribeiro’s [50] study demonstrated that alcohol consumption mainly caused high-frequency hearing loss, and the abnormality in transient evoked otoacoustic emissions in drinkers suggested that alcohol consumption was liable to damage outer hair cells. In the central nervous system, alcohol consumption reduces excitatory neurotransmission (by modulating NMDA receptors) and enhances inhibitory neurotransmission (by modulating GABA receptors) [51], and cochlear hair cells are innervated by GABA efferent neurons, which might affect the normal function of inner hair cells through this pathway [52]. Kähkönen et al. [53] used neuromagnetic combined magnetoencephalography (MEG) and electroencephalography (EEG) study found that alcohol exerts widespread effects on the processing of sound and frequency changes, alcohol impairs sound and deviance detection bilaterally at the auditory cortex and involuntary attention shifting.

Interestingly, when we classified the study subjects by age, we found that alcohol consumption did not have a significant effect on hearing loss in the elderly, and the same finding was found for age-related hearing loss among the various types of hearing loss. Alcohol consumption had a positive correlation with noise-induced hearing loss, but there was no significant difference in age-related hearing loss. In a retrospective cohort study in China, the combined effect of alcohol and noise was found to cause a greater risk of hearing loss (9.662) than the combination of the two independent variables: alcohol and noise (7.996), indicating that there may be synergistic effects of noise and alcohol consumption [34]. However, the biological mechanisms underlying the synergistic effects of noise exposure and alcohol consumption on hearing loss remain unclear. The specific reason may be related to the damage of the inner ear blood vessels, cochlear vestibular organs, and auditory nerves by both noise and alcohol consumption [54, 55]. However, age-related hearing loss could be influenced by alcohol consumption, involving several underlying mechanisms such as impairment of cochlear blood supply, resulting in hypoxia and ischemic damage, oxidative stress and associated mitochondrial dysfunction, loss of neurosensory cochlear cells, and neurodegeneration of the central auditory pathways [56, 57]. However, moderate alcohol intake may be protective against cochlear blood flow [58], promoting cytoprotection, and directly enhancing neuroprotective mechanisms that preserve hearing [59]. By contract, alcohol intake may also adversely alter the central processing of auditory information [55, 56].

To properly interpret the results of the present study, it is necessary to consider several limitations. First, there was no standard definition of alcohol consumption, which was generally a subjective answer to the questionnaire. Some articles were classified by the type of alcohol consumed, others by the amount of alcohol consumed (and in different units of measurement), or simply as two categories, drinking and non-drinking. Due to the limited number of articles and the mixed classification methods, this study classified drinking into two categories: drinking and non-drinking (in this study, in the case of multiple classifications, the group with maximum alcohol consumption was considered as the drinking group). Second, the diagnostic criteria for hearing loss were inconsistent among the included studies. Some chose the average value in the high-frequency type (3,4,6 Hz) as the standard hearing loss threshold, and some were based on the speech-frequency type (0.5,1,2 Hz). Moreover, the hearing thresholds varied, which might have led to more heterogeneity. Third, to measure hearing acuity precisely, control of ambient noise level, the examiners’ techniques, and the precision of the audiometer are important for good reproducibility. Although most of these studies described one or two of these important factors, the overall adjustment for these issues might not be sufficient. Finally, we did not examine the relationship between different alcohol dose levels and the risk of hearing loss because there was insufficient information about the dose-response relationship in this study.

Additional rigorous mega-cohort studies or large-scale randomized trials on the association between alcohol consumption and hearing loss are urgently required. Mega-cohort studies involving alcohol consumption habits are realistic and preferable but require several improvements. First, drinking behavior requires a standardized definition that includes at least three aspects: frequency of drinking, quantity of drinking per day, and episodes of heavy drinking. Fortunately, as early as 1993, the WHO developed and released a drinking questionnaire designed to quickly and easily detect drinkers with risky drinking patterns [60]. Subsequently, in 1998, Bush et al. [61] developed a more concise drinking questionnaire (AUDIT-C), which proved to be more representative and easier to use while screening large populations. The AUDIT-C is a user-friendly instrument that should be routinely included in epidemiological assessments, as it quantifies the frequency of drinking, the usual quantity of alcohol consumed per day, and the frequency of alcohol abuse. This is a good quantitative and uniform standard for future evidence of alcohol consumption. While large cohort studies are not easy to implement, we encourage rigorous implementation of pure tone audiometry rather than questionnaires to investigate hearing loss. Pure tone audiometry provides a more accurate estimate of whether the problem is in the left or right ear, whether hearing loss is high-frequency or speech-frequency, and the severity of hearing loss.

In conclusion, our systematic review and meta-analysis of available epidemiological studies found a positive association between alcohol consumption and hearing loss, suggesting that alcohol consumption may increase the risk of hearing loss. As some inherent limitations in the original studies were observed, no definitive conclusion could currently be drawn. We recommend that more well-designed prospective studies are needed to confirm the relationship between intake of alcohol and risk of hearing loss.

## Supporting information

S1 Table PRISMA 2020 checklist. (DOCX)

S2 Table Detailed search strategy. (DOCX)

S1 Fig Funnel plot with included 18 studies. (TIF)
