# Supplementary material for: Alcohol as a risk factor for hearing loss: A systematic review and meta-analysis
Source: PLoS One. 2023 Jan 20;18(1):e0280641. doi: 10.1371/journal.pone.0280641 (PMC9858841; doi:10.1371/journal.pone.0280641)
Supplement: S2 Table — (DOCX) [file pone.0280641.s002.docx]

**Table S2** Detailed search strategy

| #1 | Search"Hearing Loss"[Mesh] | 70651 |
| --- | --- | --- |
| #2 | Search (((((((((((Loss, Hearing) OR Hypoacusis) OR Hypoacuses) OR Hearing Impairment) OR Deafness, Transitory) OR Deafnesses, Transitory) OR Transitory Deafness) OR Transitory Deafnesses) OR Transitory Hearing Loss) OR Hearing Loss, Transitory) OR Loss, Transitory Hearing) OR Transitory Hearing Losses | 1105484 |
| #3 | Search "Alcohol Drinking"[Mesh] | 70843 |
| #4 | Search ((((((((Drinking, Alcohol) OR Alcohol Drinking Habits) OR Alcohol Drinking Habit) OR Drinking Habit, Alcohol) OR Drinking Habits, Alcohol) OR Habit, Alcohol Drinking) OR Habits, Alcohol Drinking) OR Alcohol Consumption) OR Consumption, Alcohol | 126373 |
| #5 | Search ("Hearing Loss"[Mesh]) OR ((((((((((((Loss, Hearing) OR (Hypoacusis)) OR (Hypoacuses)) OR (Hearing Impairment)) OR (Deafness, Transitory)) OR (Deafnesses, Transitory)) OR (Transitory Deafness)) OR (Transitory Deafnesses)) OR (Transitory Hearing Loss)) OR (Hearing Loss, Transitory)) OR (Loss, Transitory Hearing)) OR (Transitory Hearing Losses)) | 105484 |
| #6 | Search ("Alcohol Drinking"[Mesh]) OR (((((((((((Drinking, Alcohol) OR (Alcohol Consumption)) OR (Consumption, Alcohol)) OR (Alcohol Intake)) OR (Alcohol Intakes)) OR (Intake, Alcohol)) OR (Alcohol Drinking Habits)) OR (Alcohol Drinking Habit)) OR (Drinking Habit, Alcohol)) OR (Habit, Alcohol Drinking)) OR (Habits, Alcohol Drinking)) | 126373 |
| #7 | Search: (("Hearing Loss"[Mesh]) OR ((((((((((((Loss, Hearing) OR (Hypoacusis)) OR (Hypoacuses)) OR (Hearing Impairment)) OR (Deafness, Transitory)) OR (Deafnesses, Transitory)) OR (Transitory Deafness)) OR (Transitory Deafnesses)) OR (Transitory Hearing Loss)) OR (Hearing Loss, Transitory)) OR (Loss, Transitory Hearing)) OR (Transitory Hearing Losses))) AND (("Alcohol Drinking"[Mesh]) OR (((((((((((Drinking, Alcohol) OR (Alcohol Consumption)) OR (Consumption, Alcohol)) OR (Alcohol Intake)) OR (Alcohol Intakes)) OR (Intake, Alcohol)) OR (Alcohol Drinking Habits)) OR (Alcohol Drinking Habit)) OR (Drinking Habit, Alcohol)) OR (Habit, Alcohol Drinking)) OR (Habits, Alcohol Drinking))) | 200 |

Take pubmed as an example.
