# Supplementary figures and images for: Alcohol as a risk factor for hearing loss: A systematic review and meta-analysis
Source: PLoS One. 2023 Jan 20;18(1):e0280641. doi: 10.1371/journal.pone.0280641 (PMC9858841; doi:10.1371/journal.pone.0280641)

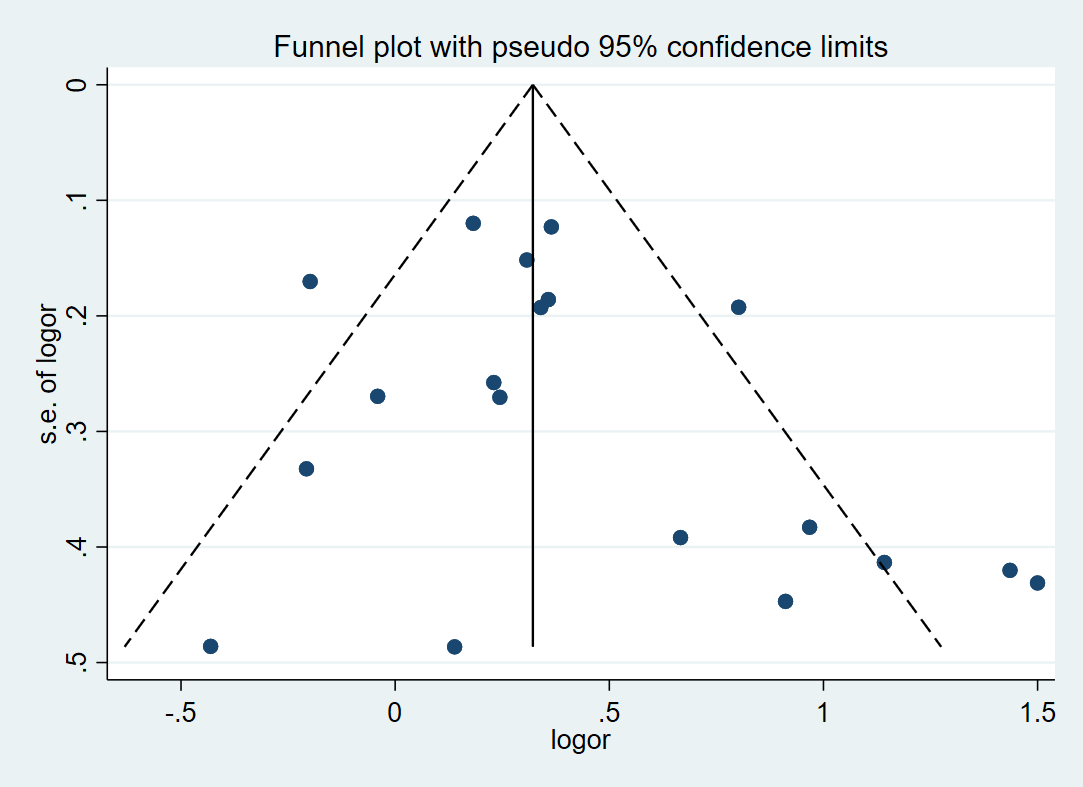

Supplement: S1 Fig — (TIF) [file pone.0280641.s003.tif]
